# Signal and reward in wild fleshy fruits: Does fruit scent predict nutrient content?

**DOI:** 10.1002/ece3.5573

**Published:** 2019-08-22

**Authors:** Omer Nevo, Diary Razafimandimby, Kim Valenta, Juan Antonio James Jeffrey, Christoph Reisdorff, Colin A. Chapman, Jörg U. Ganzhorn, Manfred Ayasse

**Affiliations:** ^1^ Institute of Evolutionary Ecology and Conservation Genomics Ulm University Ulm Germany; ^2^ Faculty of Sciences, Zoology and Animal Biodiversity University of Antananarivo Antananarivo Madagascar; ^3^ Department of Anthropology University of Florida Gainesville FL USA; ^4^ Department of Molecular and Cell Biology University of Connecticut Storrs CT USA; ^5^ Institute of Plant Science and Microbiology University of Hamburg Hamburg Germany; ^6^ Department of Anthropology McGill University Montreal QC Canada; ^7^ School of Life Science University of KwaZulu‐Natal Scottsville South Africa; ^8^ Key Laboratory of Resource Biology and Biotechnology in Western China of Ministry of Education College of Life Science Northwest University Xian China; ^9^ Animal Ecology and Conservation University of Hamburg Hamburg Germany

**Keywords:** animal–plant interactions, communication, frugivory, olfaction, seed dispersal

## Abstract

1. Plant species with fleshy fruits offer animals rewards such as sugar, protein, and fat, to feed on their fruits and disperse their seeds. They have also evolved visual and olfactory signals indicating their presence and ripeness.
2. In some systems, fruit color serves as a reliable visual signal of nutrient content. Yet even though many volatile chemicals used as olfactory signals derive from nutrients animals seek, it is still unknown whether fruit scent encodes information regarding nutrient content in wild fruits.
3. We examine the relationship between olfactory signals and nutrient rewards in 28 fruiting plant species in Madagascar. We measured the relative amounts of four chemical classes in fruit scent using gas chromatography and mass spectrometry, as well as the relative amounts of sugar and protein in fruit pulp.
4. We found that protein levels are not associated with elevated amounts of chemically related volatile compounds in fruit scent. In contrast, sugar content is strongly associated with the chemical composition of fruit scent.
5. To our knowledge, this is the first research to explore the connection between fruit chemical signals and nutrient rewards. Our results imply that in the case of sugar, fruit scent is predictive of nutrient content and hence an honest signal.

## Graphical abstract

Fruits have evolved various signals and rewards to attract animals to consume them and disperse their seeds. This study examines the relationship between chemical signal and reward—scent and macronutrients, and suggests that fruit scent signifies the amount of some nutrients in fruits.

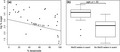

## 1 INTRODUCTION

Fleshy fruits have evolved multiple times across angiosperm families (Bolmgren & Eriksson, 2005, 2010), and dispersal by fruit‐eating animals is the predominant seed dispersal strategy among tropical woody plants (Howe & Westley, 1988). Excluding a few cases of mimetic fruits that do not offer a reward (Galetti, 2002), fleshy fruits obtain animal seed dispersal services by offering macronutrients such as sugar, fat, and protein, as well as water, antioxidants, minerals, and vitamins (Contreras‐Calderón, Calderón‐Jaimes, Guerra‐Hernández, & García‐Villanova, 2011; Herrera, 1984; Howe & Estabrook, 1977). In many lineages, fruit traits such as size (Brodie, 2017; Galetti et al., 2013), shape (Lord, 2002), and husk thickness (Janson, 1983) are suggested to have evolved to increase the accessibility and attractiveness of fruits to frugivores (Valenta, Nevo, Martel, & Chapman, 2017). In addition to characteristics which directly enhance fruit quality from the perspective of the frugivore, some fruit traits have evolved as signals which help animals detect, identify, and select ripe fruits. Fruits whose seeds are dispersed by different animals tend to be differently colored (Lomáscolo & Schaefer, 2010; Valenta et al., 2018), and there is evidence that fruit color is under selection to increase color contrasts with background foliage to render fruits more visually conspicuous (Lomáscolo & Schaefer, 2010; Nevo, Valenta, et al., 2018; Schaefer, Valido, & Jordano, 2014). Similarly, fruit scent—the volatile chemicals emitted by ripe fruits—plays a role in frugivore–plant interactions (Nevo & Ayasse, 2019). Ripe fruit scent facilitates bat (Hodgkison et al., 2013, 2007) and primate (Melin et al., 2019; Nevo et al., 2015; Nevo & Heymann, 2015; Nevo, Razafimandimby, Jeffrey, Schulz, & Ayasse, 2018; Nevo & Valenta, 2018; Valenta et al., 2013) fruit selection, and the ripeness of fruits is signaled by the chemical composition of fruit scent (Hodgkison et al., 2007; Nevo, Heymann, Schulz, & Ayasse, 2016; Nevo, Razafimandimby, et al., 2018). In this, fruit scent is now recognized to play an important role in animal–plant interactions, similar to the more thoroughly studied role of floral scent in mediating pollinator–plant interactions (Raguso, 2008; Schiestl, 2015).

Whether visual and olfactory signals go beyond the signaling ripeness/presence and provide information on nutrient content is still debated (Albrecht, Hagge, Schabo, Schaefer, & Farwig, 2018). Studies of fruit color have predominated research on this debate. In the black elder (*Sambucus nigra*), visual contrasts in the pedicels bearing the fruits are positively associated with sugar content (Schaefer & Braun, 2009). Chromatic and achromatic (brightness) properties of fruits are also sometimes correlated with nutritional content (Cazetta, Galetti, Rezende, & Schaefer, 2012; Valido, Schaefer, & Jordano, 2011). In Mediterranean habitats, fruit color correlates with lipid content (Schaefer et al., 2014), and in northern Europe, fruit brightness predicts lipid, sugar, and anthocyanin content (Albrecht et al., 2018). Interestingly, the relationship between lipid content and brightness is inverted in the latter two studies (Albrecht et al., 2018; Schaefer et al., 2014), suggesting that color signals may be location dependent and do not reflect an inherent biochemical connection between signal and reward. The absence of biochemical associations between signal and reward renders the evolution of honest signals less likely and would require either repeated interactions that allow frugivores to punish dishonest mutualists or a costliness of signals (Schaefer & Ruxton, 2011).

The study of fruit scent has lagged far behind that of fruit color, and little is known about whether scent is predictive of nutritional content (Nevo & Ayasse, 2019; Nevo & Valenta, 2018). Most investigations of fruit scent have estimated overall amounts of VOCs emitted and did not identify their chemical constituents (Lomáscolo, Levey, Kimball, Bolker, & Alborn, 2010; Valenta et al., 2015, 2013), or have focused on summarizing indices based on entire bouquets rather than individual chemicals (Nevo et al., 2016; Nevo, Razafimandimby, et al., 2018). Ethanol, a product of sugar fermentation, has been suggested to offer a reliable cue for sugar content in fruits (Dudley, 2000, 2002), and while it is correlated with sugar level in some fruits (Dominy, 2004; Sánchez, Korine, Pinshow, & Dudley, 2004; Sánchez et al., 2006), evidence for its use by frugivores is so far absent (Nevo & Valenta, 2018). Notably, ethanol is not a plant secondary metabolite but rather a product of microbial activity. Thus, there is to date no information about whether aroma compounds synthesized by plants are indicative of fruit nutritional content.

The scent of fruit of a given species typically contains dozens to hundreds of volatile organic compounds (VOCs), including terpenoids, aromatics, fatty acid derivatives, and, more rarely, nitrogen‐ and sulfur‐containing compounds (Nevo et al., 2016; Nevo, Razafimandimby, et al., 2018; Nevo & Valenta, 2018). Many fruit VOCs are synthesized from precursors to nutritionally beneficial compounds, and thus, the presence or concentration of various VOCs may reliably convey the nutrient content of a fruit (Goff & Klee, 2006). A direct biochemical relationship between signal and reward gives a strongest basis on which honest signals can evolve (Schaefer & Ruxton, 2011). The evolution of such honest signals is expected because they can enhance fitness of both animals and plants. Under this scenario, animal dispersers can select the most nutritious fruits, and plants can benefit by deterring animals from feeding on fruits that do not have seeds ready for dispersal. In addition, offering dispersers reliable nutrient information can make fruits more attractive and give plants an advantage in attracting seed dispersers. However, to date, no study has tested whether the concentration of any VOC or class of VOCs is a reliable indicator of nutrient content in wild fruits.

Here, we examine whether fruit scent chemistry is predictive of nutrient content across 28 plant species from Ranomafana National Park, Madagascar. Previous work has shown that lemurs, the main seed dispersers in the system, rely on fruit scent to identify ripe fruits (Nevo, Razafimandimby, et al., 2018; Valenta et al., 2013). We chose four VOC classes hypothesized to be positively correlated with nutrient content: nitrogen/sulfur‐containing compounds (henceforth N/S), terpenoids, methyl/ethyl esters (Nevo & Valenta, 2018), and aromatic compounds. N/S compounds are synthesized from metabolized protein (Knudsen, Eriksson, Gershenzon, & Ståhl, 2006), and thus, their presence in fruit scent can possibly be associated with protein content. Similarly, aromatic compounds are synthesized from the amino acid l‐phenylalanine (Widhalm & Dudareva, 2015) and may therefore be more common in protein‐rich fruits. Terpenoids are ubiquitous in fruit scent (Hodgkison et al., 2013; Nevo, Razafimandimby, et al., 2018) and share a biosynthetic pathway with isoprene—a compound whose emission in leaves is associated with elevated photosynthesis (Lerdau & Throop, 2000). Methyl and ethyl esters are synthesized from fusing a carboxylic acid and methanol or ethanol, which have been suggested to be a product of fruit maturation, either of cell wall degradation (methanol) or of sugar fermentation (ethanol) (Nevo & Valenta, 2018; Sánchez et al., 2006). Our main goal is to examine whether, across species, chemical signals consistently and reliably signal fruit nutrient quality. We conducted chemical analysis of fruit nutritional content and scent, and used phylogenetically controlled models to examine whether, across species, fruits with higher concentrations of protein and sugar also emit more NS compounds, aromatic compounds, terpenoids, or methyl/ethyl esters. We further test the phylogenetic signal in all traits to examine whether closely related taxa tend to be similar and compare the nutritional content of lemur‐ and bird‐dispersed fruits.

## 2 MATERIALS AND METHODS

The project was conducted in Ranomafana National Park, Madagascar between October 2016 and December 2017. In total, 410 ripe fruits (mean: 14.6 per individual tree) of 83 individual plants (mean: 2.96 per species) of 28 plant species were collected and brought to the laboratory within 3 hr of collection for processing (Table S1 and Figure S1). Fruits were collected during the day (8–14 am). As a part of a larger project that compared ripe and unripe fruits, all fruits included in the study were fully ripe: They changed their color and softened to the degree to which ripe fruits of the species do, contained fully mature and viable seeds, and were generally in the ripeness stage in which they are consumed by frugivores. Although we could not fully standardize the sample collection (e.g., collect all fruits at “peak ripeness”), each sample is composed of multiple individual fruits, and in the vast majority of species also multiple individual plants, pooled together and averaged (Table S1). This should eliminate most of the noise, which may have been introduced during sample collection. Seventeen species in the system are consumed solely by lemurs, who are also known to relay on fruit scent to identify ripe fruits, and 11 are either exclusively or to a large degree consumed by frugivorous birds (Nevo, Razafimandimby, et al., 2018, Table S1).

### 2.1 Scent sampling and analysis

We used fruit scent data published in Nevo, Valenta, et al. (2018). We sampled scent using semistatic headspace procedure. We placed the sample in a chamber made of 40 cm of an oven bag (Toppits). The bags were completely sealed on one end. On the other, they were sealed around a teflon tube holding a chromatoprobe VOC trap (Dötterl & Jürgens, 2005). Chromatoprobes contained 1.5 mg Tenax, 1.5 mg of Carbotrap, and 1.5 mg of Carbosieve III (all from Sigma­Aldrich) trapped between layers of glass wool. After 30 min, we pumped the accumulating air in the bag for 1 min onto the trap at 200 ml/min. 1.5 hr later, the bag was emptied by pumping all air onto the same trap for 10 min. Afterward, we stored the probe at −20°C.

We analyzed scent samples on an Agilent gas chromatograph 7890B equipped with an Agilent DB­5 unpolar capillary column (DB­5, 30 m × 0.25 mm diameter; Agilent Technologies) and a cold injection system (CIS 4C; Gerstel), coupled with an Agilent mass spectrometer 5977A. Samples were introduced to the thermal desorption unit (TDU) at 10°C. After 1 min, the TDU started heating up at 15°C/min until it reached 300°C, a temperature which was held for 15 min. The liner was cooled to −100°C using liquid nitrogen. After the transfer to the liner, it was heated up with 12°C/min until the temperature reached 290°C, which was maintained for 6 min. Initial oven temperature was 50°C. This temperature was maintained for 1 min and then increased by 10°C/min to 325°C, which was held for 20 min. The MS transfer line temperature was set to 280°C, the MS source temperature was set to 230°C, and the MS quad temperature was set to 150°C. The MS operated at electron ionization mode and scanned between 35 and 450 Da.

We analyzed the samples using Amdis 2.71. We identified VOCs based on their mass spectra using the NIST11 mass spectra library and their retention indices, which were calculated using an n‐alkane reference mixture. Compounds that are known contaminants (e.g., siloxanes and phthalates) were excluded. Other contaminants that we found in control samples were also potentially genuine plant compounds. We therefore calculated their mean amount in the controls and subtracted this sum from all samples.

To calculate the relative abundance of N/S compounds, aromatic compounds, terpenoids, and methyl/ethyl esters, we summed the estimated amount of all compounds of those categories and divided them by the sum of all compounds identified in the scent bouquets. We used relative amounts of chemical compounds because it allowed direct comparison of species with fruits of varying sizes, and also because when dealing with complex scent bouquets, animals tend to perceive fragrance as mixtures rather than individual compounds (Wilson, Stevenson, & Stevenson, 2006), thus making the relative amounts of scent compounds more ecologically relevant. N/S compounds were defined as all VOCs that contain nitrogen or sulfur. Aromatic compounds include all those which contain at least one aromatic ring. Terpenoids include all monoterpenes, sesquiterpenes, and derivatives such as linalool. Methyl/ethyl esters include all volatile esters with methanol or ethanol comprising the alcohol component. Note that some compounds belong to two categories (e.g., benzoic acid, methyl ester) and are thus used in both sugar and protein analyses. As a result, the relative share of the compound classes may exceed 100% in some species.

### 2.2 Nutritional analysis

Subsequent to scent analysis, the same fruits were used for nutritional analyses. Seeds were extracted, the wet mass was determined, samples were dried at 45°C until fully dry, and dry mass was determined. The nitrogen content (a proxy of crude protein) was measured by mass spectrometry according to Tom‐Dery, Eller, Jensen, and Reisdorff (2018) in aliquots of dried samples by an elemental analyzer (EURO‐EA 3000; Euro Vector). Mass calibration was conducted by the use of the certified standard 2,5‐bis (5‐tert‐butyl‐2‐benzoxazol‐2‐yl) thiophene (6.51% N; 72.52% C; HEKAtech). Sugar content analysis followed the photometric procedures outlined by Donati, Bollen, Borgognini‐Tarli, and Ganzhorn (2007). Dried samples were ground to pass a 1‐mm sieve and kept in a desiccator prior to analyses. Soluble carbohydrates were extracted with 50% methanol. Concentrations of soluble sugars were determined as the equivalent of galactose after acid hydrolyzation of the 50% methanol extract. They should be considered as relative units rather than absolute measures.

### 2.3 Statistical analysis

In all cases where more than one plant per species was available, we pooled all samples and calculated the mean amounts for the species. Sugar levels were missing for *Chassalia ternifolia*, thus analyses that include sugar are for 27 species. All our models used some of six variables: % protein, % sugar (both from the dry weight), % N/S compounds, % aromatics, % terpenoids, and % methyl/ethyl esters (latter four from total scent bouquet). Protein, sugar, and % aromatics were log transformed to acquire distributions compatible with the statistical tests. N/S compounds and methyl/ethyl esters were highly zero‐inflated, with over a third of the species not emitting these compounds at all. Thus, we converted their values to binomial variables, noting whether NS compounds or methyl/ethyl esters are present or absent in the scent bouquet of a species.

We used two phylogenetically controlled least‐square regression models (PGLS) and a phylogeny by Zanne et al. (2014) and Brownian motion correlation structure to test whether (a) protein content in pulp is predicted by the presence of N/S compound or the relative amount of aromatic compounds, independent of phylogeny and each other; and (b) sugar levels are predicted by the presence of methyl/ethyl esters and the relative amount of terpenoids, independent of phylogeny, and each other. To verify model assumptions, we used variance inflation factors to verify that there were no collinearity issues, DFFITS for influence diagnostics, and q–q plots, histograms of the residuals and plotting the residuals versus the fitted values to verify the normality and homogeneity of the residuals. We used a similar approach to compare the protein and sugar contents of lemur‐ and bird‐dispersed species. Species were categorized as either lemur‐dispersed (exclusively lemur‐dispersed) and bird‐mixed (exclusively, or mostly bird‐dispersed, in some species, there are also records of lemurs occasionally feeding on the fruits). To further assess the robustness of the results, we calculated the phylogenetic signal in all variables. Since in all cases we found no significant phylogenetic signal (see Section 3), we also ran all analyses as linear regression models, that is, using the same variables but without controlling for phylogeny. We calculated the phylogenetic signal in nutrient content and VOC class using Pagel's Lambda (Pagel, 1999). All analyses were done using R 3.4.3 (R Core Team, 2014) using packages ape (Paradis, Claude, & Strimmer, 2004), car (Fox & Weisberg, 2011), phytools (Revell, 2012), and nlme (Pinheiro, Bates, DebRoy, Sarkar, & R Core Team, 2017).

## 3 RESULTS

Both scent and nutritional values showed strong variation, even within families (Table 1; Table S1). For example, % nitrogen varied between the congeneric *Ficus reflexa* and *Ficus politoria* (Moraceae) between 0.4% and 1.8%, respectively. Sugar levels showed even greater variance, ranging between 3.3% in *Cryptocaria* sp. (Lauraceae) and 69.7% in *Oncostemum botryoides* (Primulaceae) (Table 1; Table S1). Although the small sample size within species did not allow quantitative analysis of within‐species variance, species did not show much variance, and especially in terms of scent, fruits from different individuals tended to be dominated by the same chemical compounds.

**Table 1:** Scent and nutritional data for all species

	Nutrition		Scent			
	% nitrogen (mean absolute amount in a single fruit, mg)	% sugar (mean absolute amount in a single fruit, mg)	% aromatics	% terpenoids	% N/S	% methyl/ethyl esters
Anacardiaceae						
*Micronychia macrophylla*	1 (0.9)	29 (28.7)	0.5	91.1	0.0	0.1
*Weinmannia rutenbergii*	0.5 (0)	6.8 (0.6)	2.3	33.6	6.4	0.0
Araliaceae						
*Polyscias* sp.	1.3 (3)	16.6 (51.3)	12.2	44.8	6.1	0.0
Clusiaceae						
*Garcinia* sp.	0.5 (1.3)	37.8 (116.8)	39.3	17.6	0.1	81.0
Euphorbiaceae						
*Macaranga myriolepida*	1.6 (0.3)	6.1 (1)	0.3	95.6	0.1	0.6
Hypericaceae						
*Psorospermum androsaemifolium*	1.6 (1.6)	13.2 (13.4)	8.5	77.3	0.0	0.0
Lauraceae						
*Cryptocaria crassifolia*	1.6 (2.5)	18.1 (29.4)	0.3	95.3	0.0	0.0
*Cryptocaria* sp.	0.7 (16.8)	3.3 (79.4)	0.1	97.9	0.0	0.0
Moraceae						
*Ficus botryoides*	1.5 (35)	9.8 (219.8)	4.0	62.4	0.4	25.2
*Ficus lutea*	0.6 (3.2)	25.6 (125.8)	0.7	90.5	0.3	0.2
*Ficus politoria*	1.8 (2.5)	20.4 (27)	12.1	36.7	2.9	0.4
*Ficus reflexa*	0.4 (0.2)	46.3 (25.6)	6.9	29.4	0.3	1.0
*Ficus tiliifolia*	0.8 (13.3)	31.8 (529.5)	0.1	0.9	0.0	86.1
Myrtaceae						
*Eugenia* sp.	1.2 (5.3)	3.9 (18.8)	0.5	98.3	0.0	0.0
*Psidium cattleianum*	0.5 (7.3)	40.9 (508.3)	0.6	19.3	0.1	10.6
*Syzygium emirnese*	0.7 (0.2)	56.7 (19.2)	6.4	40.2	0.2	16.2
*Syzygium parkeri*	0.6 (0.5)	26.2 (20.7)	10.3	23.6	2.0	0.0
Oleaceae						
*Noronhia incurvifolius*	0.7 (3.1)	38.7 (166.5)	0.2	13.0	0.0	0.4
Piperaceae						
*Piper* sp.	1.4 (0.2)	13.4 (1.9)	0.8	93.7	0.0	0.0
Primulaceae						
*Oncostemum botryoides*	0.5 (0.5)	69.7 (66.2)	75.6	14.6	0.0	73.1
*Oncostemum nervosum*	0.7 (0.1)	51 (8.4)	2.0	71.4	1.1	2.8
Rubiaceae						
*Chassalia ternifolia*	1.3 (0.1)		22.7	28.9	3.3	1.5
*Coptosperma* sp.	0.6 (2.3)	36.7 (142.4)	11.9	1.8	0.0	61.1
*Mussaenda arcuata*	1.4 (1.5)	23.1 (23.6)	7.5	60.2	0.2	7.4
*Mussaenda erectiloba*	0.9 (5.5)	32.6 (204.1)	2.1	94.9	0.0	0.0
*Psychotria* sp.	1.1 (1.1)	47.9 (47.9)	4.1	70.3	0.1	7.6
*Pyrostria* sp.	0.5 (0.6)	46.1 (56.5)	5.0	9.2	0.1	12.2
Rutaceae						
*Zanthoxylum madagascariensis*	1.4 (0.6)	5.4 (2.3)	1.3	96.4	0.0	0.0

Percentage terpenoids, N/S compounds, and methyl/ethyl esters in scent; percentage of nitrogen (proxy of protein content) and sugar in ripe fruit dry weight. Note that in both scent and nutrition the percentages presented here do not add up to, or exceed, 100%. In scent, the rest refers to various aromatic compounds and fatty acid derivatives. In species in which scent components exceed 100%, it is because some compounds are classified in two categories (e.g., methyl benzoate). In nutrition, the analyses do not consider other components (e.g., fat, fiber, secondary compounds). Numbers in brackets are absolute amounts of nutrients (mg) in a single fruit.

**Figure 1:**
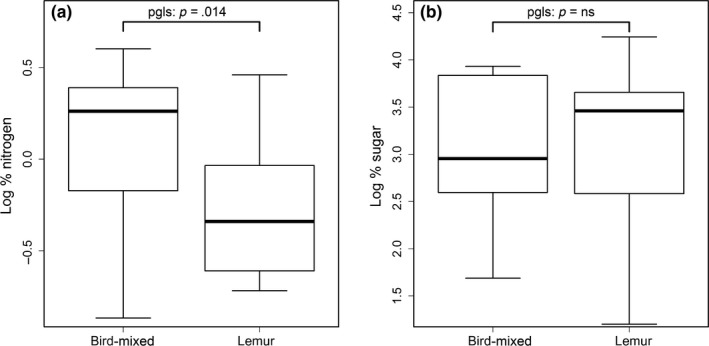
Relative amounts of sugar and nitrogen in lemur‐ and bird‐mixed consumed species. Log % sugar, log % nitrogen—log transformed percentage sugar and nitrogen in dry pulp. *N* = 28 species for protein and 27 for sugar (see Section 2 for more details). *p* Values are from a phylogenetically controlled generalized least‐squares regression model (PGLS) using he phylogeny provided by Zanne et al. (2014)

Lemur‐ and bird‐dispersed species differed in their protein, but not sugar content. Nitrogen levels were significantly higher in bird‐dispersed species (pgls: *p* = .014), but sugar content was similar (pgls: *p* = .8; Figure 1).

**Figure 2:**
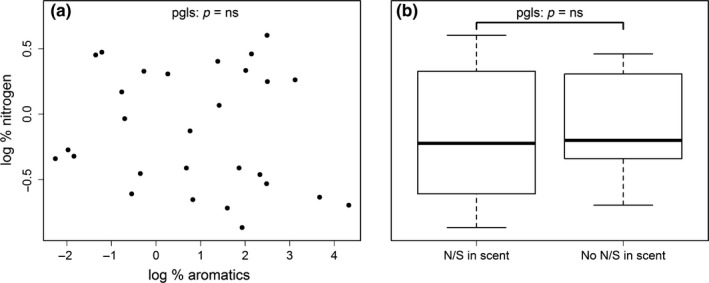
Relationship between nitrogen and the relative share of aromatic compounds (a) or (b) presence of nitrogen/sulfur (N/S) containing compounds in fruit scent. N/S—nitrogen‐ and sulfur‐containing VOCs. log % protein—percentage protein in fruit dry weight, log transformed. Log % aromatics—percentage aromatic compounds in scent profile, log transformed. *N* = 28 species. *p* Values are from a phylogenetically controlled generalized least‐squares regression model (PGLS) using a phylogeny by Zanne et al. (2014)

Contrary to our predictions, protein levels were not predicted by either the presence of N/S or the aromatic compounds. The model containing N/S and aromatic compounds did not explain the variance in % nitrogen in fruit pulp better than a null model which did not include them (likelihood ratio test: *L*. ratio = 0.74, *p* = ns; Figure 2).

**Figure 3:**
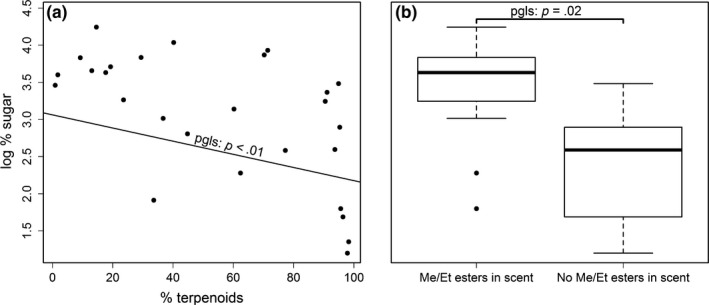
The relationship between sugar level and the relative amounts of terpenoids (a) and methyl/ethyl esters in fruit scent (b). (a): % terpenoids—relative amount of terpenoids in ripe fruit scent. (b): presence or absence of methyl and ethyl esters in fruit scent. In both: log % sugar—percentage sugar in fruit dry weight, log transformed. *Y*‐axis on the left applies to both plots. *N* = 27 species. *p* Values are from a single phylogenetically controlled generalized least‐squares regression model (PGLS) using a phylogeny by Zanne et al. (2014). Effects are thus independent of phylogeny and one another

For sugar, the full model which included % terpenoids and the presence/absence of methyl/ethyl esters explained variance in fruit sugar content significantly better than the null model (*L*. ratio = 16.14, *p* < .001). Contrary to our expectations and what the literature suggests, terpenoids in ripe fruit scent were negatively correlated with the relative amount of sugar in fruits (PGLS: *p* < .01; Figure 3a). In contrast, the presence of methyl and ethyl esters was associated with elevated sugar levels (PGLS: *p* = .02; Figure 3b). Originating from the same model, these relationships are independent of phylogeny and each other.

We found no phylogenetic signal in any of the traits measured (% nitrogen, % NS compounds, % aromatic compounds, % terpenoids, % methyl/ethyl esters: λ < .01, *p* = 1; % sugar: λ = .53, *p* = .35). Consequently, the results of linear regression models (identical to those reported above but not controlling for phylogeny) were qualitatively identical to those of the PGLS models (not shown).

## 4 DISCUSSION

Our study examined whether scent reliably and consistently signals nutrients in wild, ripe fruits. We tested whether certain chemical classes in fruit scent predict the protein concentration or sugar levels. We found that protein levels are not associated with the relative amounts of either aromatic or nitrogen/sulfur‐containing compounds, whereas sugar levels are strongly associated with the amount or presence of terpenoids and methyl/ethyl esters in fruit scent.

The absence of a clear relationship between protein levels and N/S compounds may be the result of several factors. In addition to synthesis by the plant, the presence of nitrogen‐ and sulfur‐containing compounds is likely to be affected by degradation of fruit tissue by microbes. This may in turn be influenced by the susceptibility of fruits to infestation by microbes, which is unrelated to fruit protein content. In other words, a protein‐rich yet well‐protected fruit may emit less N/S compounds than a similar, but less protected, fruit. Presumably, this and other effects may be weak enough if large amounts of protein in the pulp would result in large amounts of N/S compounds in the scent, which will mask other factors like microbial activity. Alas, protein levels in Malagasy fruits, in this study and in others (Donati et al., 2017; Ganzhorn et al., 2009; Valenta & Melin, 2012), are very low relative to fleshy fruits in other tropical systems. Not surprisingly, the scent of Malagasy fruits also tends to include fewer N/S compounds (Nevo & Valenta, 2018). Thus, paradoxically, even though in these conditions reliable signals for protein content would be highly useful for frugivores, it is possible that the low amounts of protein in fruit pulp generate a signal that is too weak to be detected by either the methods used or, possibly, frugivorous animals. A similar explanation might apply to the absence of any correlation between protein content and aromatic compounds in fruit scent.

Another possible explanation is that the absence of an association between signal and reward is linked to the lower amounts of protein found in lemur‐dispersed fruits (Figure 1). Due to their lower color vision capacities and more developed olfactory systems, lemurs tend to rely on fruit scent more than most frugivorous birds (Nevo & Ayasse, 2019). In a previous study of the system used here, only lemur‐dispersed fruits were found to signal ripeness through scent (Nevo, Razafimandimby, et al., 2018). It is thus possible that since the species which benefit more from scent signaling (lemur‐dispersed) are poor in protein, they have simply not been selected to emit reliable signals exposing their low protein content, whereas bird‐dispersed species do not do so because their target seed disperser is less likely to use olfactory cues. In other words, the relationship we expected to find would require bird‐dispersed species to emit N/S compounds, but they do not benefit from doing so.

In contrast, we found that across species sugar levels in fruits are strongly associated with chemical constituents of fruit scent. The positive association between methyl and ethyl esters and sugar levels is not surprising as alcohols are often the limiting factor in ester synthesis (Beekwilder et al., 2004). Methanol, the precursor for methyl esters, is a product of cell wall degradation, which also leads to fruit softening (Sánchez et al., 2006). Ethanol, which is used for ethyl ester synthesis, is a product of microbial fermentation, and thus, elevated sugar levels are likely to be associated with increased ethanol synthesis. Thus, sugars and methyl/ethyl esters may be highly correlated due to a straightforward biochemical pathway, and their presence in fruit scent is likely to be an honest signal for sugar content.

The negative relationship between sugars and terpenoid emission was in contrast to our expectations. We predicted a positive relationship based on the positive correlation between photosynthetic activity and isoprene synthesis in leaves (Lerdau & Throop, 2000), and the corollary inference that increased photosynthesis should be associated with both sugar and terpenoid synthesis. While terpenoids have been shown to function as frugivore attractants (Hodgkison et al., 2013; Nevo et al., 2015, 2016), many function as chemical defense barriers (Farmer, 2014; Nevo et al., 2017; Unsicker, Kunert, & Gershenzon, 2009). Thus, their synthesis and emission in ripe fruits may be dominated by factors unrelated to animal signaling. Yet crucially, the question at the core of our study was whether the presence of various chemicals in fruit scent may be consistently associated with fruit nutritional content. Even though this result is contrary to our predictions, this strong negative relationship may still be useful for frugivores.

It is important to note that the relationships reported here are *across species*, not within them. As such, the results emphasize that the presence or amount of some chemicals in fruit scent may be consistently associated with sugar content. This is the basis for honest signaling in fruit scent: If scent compounds are biochemically associated with nutrients and their presence provides the same information across species, animals can learn to use them in the context of food selection (Schaefer & Ruxton, 2011). Given these results, we predict that a similar relationship between sugar and aliphatic esters and terpenes may be present *within species*. While beyond the scope of the current study, future studies should include behavioral bioassays to examine to what extent animals prefer fruits whose scent is richer in relevant scent compounds. Yet, another possible approach to address this question is to experimentally manipulate the nutrients available for plants and record whether this affects the volatiles we hypothesized to be associated with that nutrient.

Our study focused on four chemical classes that may be predictive of sugar and protein contents. Yet, animals may seek other macronutrients such as fat or use chemical cues to avoid undesirable contents such as fiber or unpalatable secondary metabolites. Similarly, scent may also signify the presence of micronutrients such as vitamins. This could unfortunately not be addressed in the current study due to the difficulties in extracting enough plant material to conduct all the analyses, but remain an interesting avenue for future studies.

A biochemical association between signal and reward is probably the most important and common substrate on which honest signals can evolve, especially in fleshy fruits, in which the fruit is both signal and reward (Schaefer & Ruxton, 2011). To our knowledge, a relationship between chemical signals and reward has so far only been identified in specialized ant‐dispersal systems in which the attractant—often a long chain fatty acid—also serves as the reward (Pfeiffer, Huttenlocher, & Ayasse, 2010). Our results expand this phenomenon to much larger and generalized seed dispersal systems and provide the first evidence for a association between fruit chemical signals and nutrient rewards in fleshy fruits. These results suggest that fruit chemical signaling through scent is—at least in the case of sugar—constrained by their macronutrient content and thus that within species fruit scent may function as an honest signal indicating fruit quality.

## CONFLICT OF INTEREST

The authors have no competing interests.

## AUTHOR CONTRIBUTION

ON acquired funding, designed the study, collected samples, conducted fruit scent analyses, conducted statistical analyses, and wrote the manuscript. DR collected samples. KV wrote the manuscript. JAJJ helped in lab work. CAC wrote the manuscript. JUG and CR conducted nutritional analyses. MA participated in funding acquisition, project design, and writing the manuscript.

## Data Availability

All data used for the analyses are available in Table S1. Raw data from which scent variables were collected are fully available in Tables S2 and S3 in Nevo, Razafimandimby, et al., 2018. R code of statistical analysis is available at https://github.com/omernevo/Fruit-scent-and-nutrition---across-species-analysis.
