# Supplementary figures and images for: Signal and reward in wild fleshy fruits: Does fruit scent predict nutrient content?
Source: Ecol Evol. 2019 Aug 22;9(18):10534–43. doi: 10.1002/ece3.5573 (PMC6787828; doi:10.1002/ece3.5573)

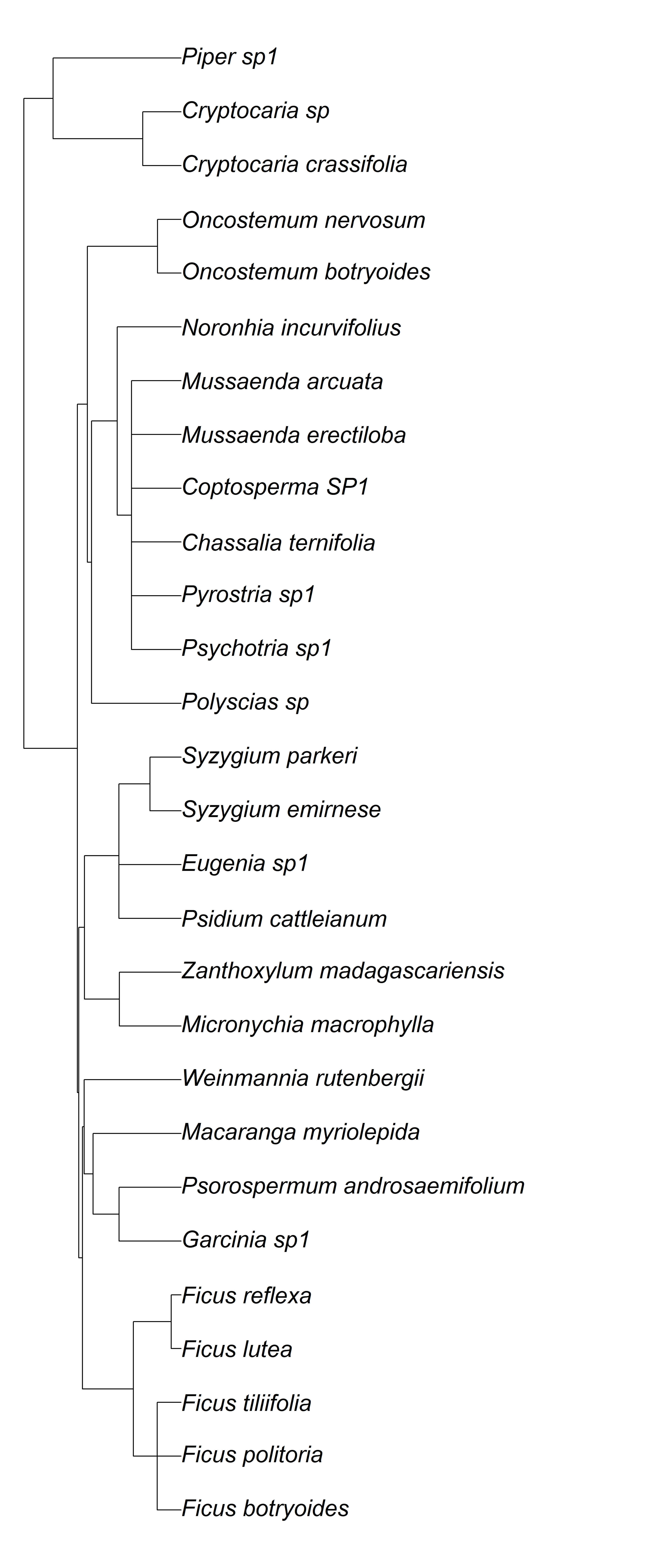

Supplement: Supplementary file 1 [file ECE3-9-10534-s001.png]
